# Zooming In, Then Out: Why We Must Apply Human-Centered Design to Transform Diabetes Technology

**DOI:** 10.1177/19322968231213665

**Published:** 2023-11-16

**Authors:** Edward C. Chao

**Affiliations:** 1 University of California, San Diego, La Jolla, CA, USA; 2 Veterans Administration San Diego Healthcare System, San Diego, CA, USA

**Keywords:** diabetes mellitus, continuous glucose monitoring, patient engagement, humanizing technology, human-centered design, diabetes technology

## Abstract

Technological advances in devices, such as continuous glucose monitors (CGMs) or intermittently scanned continuous glucose monitors (isCGMs), do not necessarily by themselves translate to improved clinical outcomes or quality of life. Human-centered design (HCD) is an accessible, flexible process that could contribute to reducing the gap between current challenges and more optimal future solutions, by continuing to refine crucial considerations, such as usability. Starting with understanding the unmet needs of patients, cultivating novel and different collaborations, and applying humility to humanize technology are three facets underlying this approach. Human-centered design can help expand our perspective to serve as another essential tool to help further refine diabetes technology.

To advance diabetes technology, we must first step back, and look around. Human-centered design (HCD) can facilitate developing more seamless, inviting, and engaging devices. Studies have demonstrated that technology has significantly improved clinical outcomes for patients living with diabetes (PWD): for type 1 diabetes (T1DM) on both continuous glucose monitors (CGMs)^1,2^ and intermittently scanned CGMs (isGCMs) (also termed flash monitors)^3^; CGM for type 2 diabetes (T2DM)^4^; and diabetes-specific quality of life measures for participants with T1DM.^5^ Yet, paradoxically, uptake remains suboptimal. Setting aside the vexing challenges with access to CGM, flash monitors, and insulin pumps, especially for disadvantaged individuals—cost and insurance coverage in the United States—barriers persist for many. Uptake can be a concern, even in nations offering subsidies. The most recently available data reveal that of 1662 patients in the T1 Exchange Registry, 27% to 41% of PWD discontinue CGMs- within one year of starting,^6^ and 12.5% of those using isCGM- discontinue after one year.^7^ What are some of the other causes of decreased satisfaction with devices?

The HCD process (Figure 1) starts with empathy: deeply understanding the patients’ needs.^8-10^ We may be confident we are well-versed on the problem, but may later learn that this is not the case. After defining needs, design thinkers question their assumptions to create a wide range of ideas, some of which will inform low-, then high-fidelity, prototypes. Multiple rounds of testing of these will elicit feedback, which will help shape what will ultimately be the final product.

**Figure 1.**
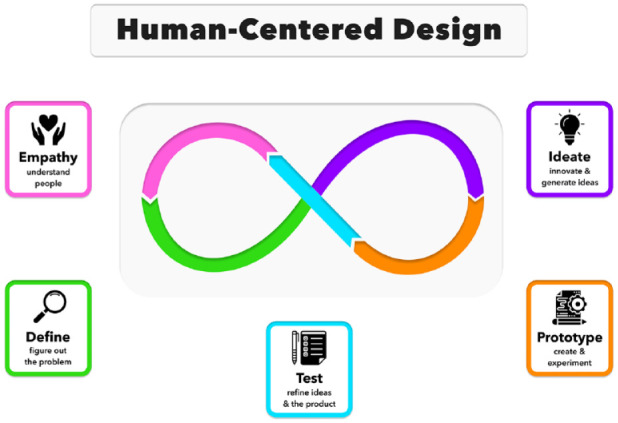
An illustration of the five steps of the human-centered design process based on design thinking.^11^ Please note that these steps can be carried out concurrently, and subsequent stages can help refine earlier steps. Figure courtesy of Ashley DuBord. Reproduced from Chao et al.^12^

HCD expands perspective. To humanize technology, we must start with the individual using the device daily, involve them at all stages of development, and expand our efforts to include improving their experience with devices; widen the team of collaborators; and humanize technology through humility.

1. **“Listen to your patient; he is trying to tell you the diagnosis.”** Dr William Osler’s wise words remind us that we must begin with the PWD, who are the most highly attuned to the pitfalls and pain points of DM technology. Tanenbaum and colleagues queried 1498 individuals in the T1D Exchange about their views of diabetes technology and found a spectrum of five groups.^13^ Surprisingly, the individuals with the most barriers and distress (3%) were the youngest. Around 54% (the d-Embracers) had the most positive views and reported the least challenges with device use. Obstacles included: not trusting diabetes technology; disliking the look of devices; feeling that using a device presented hassle; and wanting to avoid drawing attention by wearing a device. IDEO, a design and innovation company, gleaned insights from observing and speaking with PWD, and even individuals working in similar industries, to introduce changes (such as tattoo artists, who extensively use hand-held tools) to insulin pens, including discreet and “nonmedical” design; decreasing the amount of force to inject insulin; and being able to move a dial backwards if one mistakenly passed the correct dose.^14^ While starting with the patient is essential, we must view him or her as interacting with their device within a larger context: amidst the multiple, competing daily demands of diabetes, as well as work, school, family, among others. They can also reveal the workarounds that they may have crafted that may suggest future improvements. It is not just the device; it is the entire experience.
2. **Smashing silos can yield unexpected insights from unusual sources.** Just as health care depends on a well-functioning, multidisciplinary team to best help patients, research and development must also draw from the varied observations, knowledge, ideas, and experiences of multiple, diverse individuals. We must reach across traditional departmental and institutional boundaries that may isolate disciplines. The UCSD Diabetes Design Initiative (www.ddi.ucsd.edu) brings together PWD, physicians, CGM device manufacturers, cognitive science students who are learning HCD, HCD experts, and community advocates. Examples include an interactive, visual educational tool, InsuLearn (http://ddi.ucsd.edu/learn/about.html); a project on the role of patient trust in diabetes technology; and working with Dexcom to aid patients with troubleshooting. Chris Messina in 2007 proposed using hashtags to better sort social media posts and facilitate group discussions. He was initially met with skepticism, though hashtags have since become ubiquitous. He is an example of an extreme user—those who are enthusiastic and may have unique needs, and thus, insights that many may not have. Gleaning and implementing these could lead to more inclusive design that takes into account more individuals, and not just the “average” user.
3. **Humility to help humanize technology:** Health care can seem formulaic, sterile, and impersonal. Twenty-minute clinic visits can feel rushed. Telemedicine and remote monitoring may also unwittingly diminish the human element. Our patients bring to their appointments a wealth of data: glucose readings, A1c, lipids or BP. With a click or a swipe, technology can help us efficiently collect and sort this mountain of data. We must remember the unique individual humans behind the numbers, and connect with them. Humility is the key to being an outstanding clinician. Applying this quality to research is equally critical. We must be open to rethinking what we know or are used to, continue to learn, and ask new questions.

Technology can outpace our ability to thoughtfully and proactively steer, harness, and refine it. We must keep at the forefront vital considerations, including data security; how HCD could help PWD and HCP best manage and interpret CGM and isCGM data; and sustainability of devices. Artificial intelligence (AI) importantly helps and will continue to have a significant impact on health care and other facets of life. HCD offers a flexible, accessible framework that can guide us in our efforts to keep humans at the center, to best engage with AI, as well as future technological breakthroughs While HCD is certainly not a one-size-fits-all panacea, I am confident that HCD will help us strike a balance between cutting-edge advances and a humane outlook—the best of both the science and art of medicine—to continue improving diabetes technology for all involved: patients, caregivers, and health care professionals.

The most cutting-edge device serves no one if it sits on a shelf, unused. HCD must be part of an effort that cuts across traditional boundaries to unite the patient, the caregiver, the designer, the engineer, - the physician, and health care team members. To arrive at new solutions, we must continue cultivating curiosity, viewing the current landscape with fresh eyes. We must be open to experimenting, learning from these efforts, and implementing technology that is worthy of our patients’ resilient daily efforts. We must recast what technology can do and move beyond helping collect information, or providing reminders, to include facilitating engagement. Human-centered design embodies the excitement of discovery. Together with our patients, using the diverse insights we have gathered, let us take the next step to continue to refine the unending path to help them thrive.
